# Insights Into Genetic Variations of the *OCT1* Gene in Metformin Poor Responders Among Bangladeshi Type 2 Diabetic Patients

**DOI:** 10.1155/adpp/8568658

**Published:** 2025-01-29

**Authors:** Rokeya Begum, Arindita Das, Md. Jahangir Alam, Gazi Nurun Nahar Sultana

**Affiliations:** ^1^Genetic Engineering and Biotechnology Research Laboratory, Centre for Advanced Research in Sciences (CARS), University of Dhaka, Dhaka 1000, Bangladesh; ^2^Department of Genetic Engineering and Biotechnology, University of Dhaka, Dhaka 1000, Bangladesh; ^3^Department of Biochemistry, Primeasia University, Banani, Dhaka 1213, Bangladesh

**Keywords:** metformin, mutation, *OCT1* gene, side effects, Type 2 diabetes mellitus

## Abstract

Metformin is the most widely prescribed drug for the treatment of Type 2 diabetes mellitus (T2DM), but its response varies from person to person. This study aims to analyze the complete mutation spectrum of the *OCT1* gene in metformin poor responders and to explore the potential pathogenic effects of the identified mutations. Clinical features of 56 Bangladeshi T2DM patients (who showed altered response to metformin) were analyzed, and genomic DNA was extracted from their blood samples. Subsequently, the entire exons (1–11), along with flanking introns of the *OCT1* gene were amplified and sequenced. Molecular consequences of the identified mutations on OCT1 protein activity were determined through in silico analyses. In this study, 29 mutations of the *OCT1* gene were identified; among which 5 mutations (c.412-86G>T, c.970G>C, c.1386-3088_1386-3083delGAATCA, c.1498+66G>T, and c.1653C>A) were novel. It was found that nsSNPs c.181C>T, c.1022C>T, c.493G>T, c.1207A>G, and c.970G>C (novel) as well as frameshift deletions have potential deleterious effects on OCT1 protein stability and function. Some of these mutations also cause alternative splicing, as per the HSF tool. In addition, alteration of interatomic bonding in the OCT1 protein due to two high-risk mutations (c.181C>T and c.1022C>T) was found from web-based analysis. The mutations, as mentioned earlier, are the most probable causative factor of decreased metformin effectiveness and adverse side effects in T2DM patients who are poor responders. Understanding the *OCT1* gene variations of patients can help tailor treatment strategies for optimal metformin response or identify alternative medications.

## 1. Introduction

Diabetes is the seventh leading cause of death globally and a significant contributor to expensive and debilitating complications, including heart attacks, strokes, renal failure, vision loss, and lower limb amputations [1]. Type 2 diabetes mellitus (T2DM) accounts for around 90% of all cases of diabetes [2]. In the long term, T2DM is difficult to treat effectively without drugs [3]. Metformin is the most widely prescribed drug for the treatment of Type 2 diabetes because of its efficacy, safety profile, and low cost [4]. Through its extensive use, metformin has variable responses from individual to individual. If metformin does not respond sufficiently, it may cause several gastrointestinal adverse effects, including nausea, diarrhea, vomiting, bloating, dyspepsia, abdominal pain or cramps, and/or changes in intestinal motility and may lead to life-threatening lactic acidosis [5, 6].

Numerous studies showed that variations in metformin response among individuals are primarily attributed to organic cation transporters (OCTs) rather than drug-metabolizing enzymes and drug receptors [5]. OCT1 is expressed on the basolateral membrane of hepatocytes, cytoplasm of the enterocytes, and distal tubules in the kidney [7, 8]. The major function of OCT1 is the absorption, distribution, and excretion of metformin [9].

The solute carrier family 22 member 1 (*SLC22A1*) gene—commonly known as *OCT1* gene—is located on human chromosome 6q25.3. It contains 11 exons and spans over 35 kb of the genome [10, 11]. It encodes OCT1 protein which is composed of 554 amino acids. This protein is a plasma integral membrane protein. Two transcript variants coding for distinct isoforms have been identified for this gene; however, only the longer variant is responsible for encoding a functional transporter. This gene is highly polymorphic and leads to differences in transporter function, presumably by decreasing or increasing the hepatic uptake of the drug [5, 12].

The distribution of *OCT1* polymorphism and its therapeutic effect varies in ethnically diverse populations [13]. In Caucasians, a total of 25 single nucleotide polymorphisms (SNPs) of the *OCT*1 gene were identified, with three (R61C, C88R, and G401S) of them demonstrating decreased transport capabilities [14]. However, genetic variants of *OCT1* (i.e., S14F, R61C, S189L, G220V, G401S, 420del, and G465R) decreased metformin uptake in populations with European ancestries but not in Asian American, Chinese, Korean, and Japanese populations [15]. According to another study, two nsSNPs (P283L and P341L) of the *OCT1* gene identified in the Japanese population and Korean population—with allele frequencies of 1.3% and 16.7%, respectively—exhibited reduced transport activity [9, 16, 17]. In the Indian population, these two SNPs were also identified [18, 19]. Another study showed that intronic 1386A>C showed a 5.6 times greater chance of responding to metformin treatment in the South Indian population, but there was no significant relationship with HbA1c levels in the Danish population [20, 21].

Since the occurrence of *OCT1* genetic variation and its impact on metformin treatment efficacy differs among different populations, researching this topic is imperative to understand its implications for the Bangladeshi population. However, no studies in this area have been performed on the Bangladeshi population so far. Addressing this gap in research can provide valuable insights into optimizing metformin therapy for T2DM patients in Bangladesh. Therefore, in this study, we sequenced the entire coding exons (1–11) and the flanking intron sequences of the *OCT1* gene and analyzed the mutations in the *OCT1* gene of unrelated Bangladeshi T2DM patients who are poor responder of metformin. Besides, we utilized several in silico tools to explore the molecular consequences of these mutations on OCT1 protein activity, stability, and mRNA splicing.

## 2. Methods and Materials

### 2.1. Study Population

In this study, 56 T2DM patients (enrolled in BIRDEM General Hospital of Bangladesh) from Bangladeshi families who were metformin poor responders were included. The selection criteria were that they had been using metformin for at least 7 months to manage blood glucose levels and taking additional medications to complement it, including gastric medications for those experiencing gastrointestinal side effects from metformin [22]. Written consent was obtained from all participants before the start of this study, and this protocol was approved by the Institute Ethics Committee of BIRDEM General Hospital (BIRDEM/Academy/2017/35). All of the T2DM patients were diagnosed as having fasting plasma glucose (FPG) ≥ 7.0 mmol/L and/or postprandial plasma glucose (PPG) ≥ 11.1 mmol/L according to the diagnosis criteria of the World Health Organization in 1997.

### 2.2. Relevant Data and Sample Collection

The subjects completed a questionnaire regarding their clinical features and medication. Statistical analyses were performed to calculate the mean and standard deviation of the data using Microsoft Excel. Approximately 3.0 mL of blood sample was collected in an EDTA-coated vacutainer from patients. Collected samples were stored at −20°C until analysis and DNA extraction.

### 2.3. DNA Extraction and Polymerase Chain Reaction (PCR) Amplification

Using the FlexiGene DNA Kit (51206, QIAGEN), genomic DNA was extracted from the collected whole blood samples according to the manufacturer's protocol. The quality and quantity of extracted DNA were measured by a NanoDrop 2000 spectrophotometer and visualized by 0.8% agarose gel electrophoresis in 1 × TAE buffer. The entire coding exons (1–11) and the flanking intron sequences of the *OCT1* gene were amplified by PCR using lab-designed primers (Table 1). The PCR thermal program began with an initial denaturation at 95°C for 5 min. This was followed by 35 cycles, each consisting of 1 min at 95°C, 1 min at 58°C, and 1 min at 72°C. The thermal program concluded with a final elongation step at 72°C for 10 min. Successful amplification was confirmed by agarose gel electrophoresis and then purified using FavorPrep GEL/PCR Purification Kit (FAGCK 001, Favorgen Biotech Corp.).

### 2.4. Entire OCT1 Coding Region Mutation Detection and Genotype Analysis

The purified amplicons were cycle sequenced with the BigDye terminator v3.1 sequencing kit (4337455, Applied Biosystems). The capillary electrophoresis and subsequent analysis were performed with the ABI 3130 Genetic Analyzer (Applied Biosystems). The mutations in sample sequences of patients were detected by using NCBI BLAST (bl2seq) tools with the normal human *OCT1* sequence as a reference (obtained from the National Center for Biotechnology Information [NCBI], Reference Sequence: NC_000006.12 (160121808-160160590)). Each mutation was reconfirmed by second-time sequencing, and its homozygous and heterozygous genotypes were identified by visual inspection of chromatograms. Using genotype data of the identified SNPs, statistical analysis was performed to determine whether the study population was in Hardy–Weinberg equilibrium (HWE). In this study, genotype frequency data of the Bangladesh population in the 1000 Genomes Project (http://www.1000genomes.org/page.php) were used as healthy control data.

### 2.5. Assessment of Deleterious Effects of the Mutations

To identify novel mutations in the *OCT1* gene sequences, the NCBI database dbSNP and the Human Gene Mutation Database were used as reference sources [23, 24]. ExPASy translate tool was used to convert nucleotide sequences into corresponding amino acids. In this study, those identified mutations were considered pathogenic whose nucleotide changes resulted in nonsynonymous, frameshift, or aberrant splicing. SIFT, PolyPhen-2, MutationTaster, PhD-SNP, SNAP2, PMut, and PROVEAN web-based tools were used to evaluate the potential pathogenicity of novel and reported nonsynonymous mutations [25–31]. These tools use different algorithms and data for analyses. For instance, SIFT predicts the deleterious effect of nonsynonymous mutations based on sequence homology and physiological properties of amino acids, whereas PolyPhen-2 predicts the same thing based on evolutionary and structural considerations [25, 26].

In this study, those nsSNPs were considered potentially pathogenic if they were predicted to be deleterious by at least four of the abovementioned in silico tools. In addition, to check the protein stability of identified mutations in this study, MUpro, INPS-MD, I-Mutant2.0, DUET, SDM, mCSM, and DynaMut tools were used [32–38]. Moreover, the effect of the deep intronic and coding region's mutations on pre-mRNA splicing was predicted by HSF [39]. Furthermore, the DynaMut webserver was used to detect potential changes in interatomic bonding within the encoded protein as a result of mutations. InterVar (https://wintervar.wglab.org/evds.php), a bioinformatics software tool, was used for the clinical interpretation of identified genetic variants in the study population by the ACMG/AMP 2015 guideline.

## 3. Results

### 3.1. Clinical Data Analysis

In this study, 56 T2DM patients (metformin poor responders) were involved. The average age of the patients was 48.07 ± 10.69. These patients were taking metformin for 3.62 ± 2.97 years to manage their blood glucose levels along with additional supplementary medications. Of them, 14 individuals (28%) were male and 36 individuals (72%) were female, and their average body mass index (BMI) was 26.10 ± 3.55 which was higher than the normal range. Their average FPG was 7.28 ± 1.98, PPG was 10.02 ± 2.76, and average glycated hemoglobin (HbA1c) was 8.13 ± 2.27 which was slightly higher than the normal range (Table 2). On the other hand, their lipid profile, except for triglyceride (TG) and low-density lipoprotein (LDL), was within range (Table 2). All of them are nondrinkers.

### 3.2. Mutation Identification

From the sequence analysis of all exons (1–11) of the target gene *OCT1* and their flanking introns, 5 novel mutations (c.412-86G>T, c.970G>C, c.1386-3088_1386-3083delGAATCA, c.1498+66G>T and c.1653C>A) along with 24 previously reported mutations were identified in this study (Table 3). From the mutation type analysis, it was found that 17.24% are deletion mutations, 37% are transversion substitution mutations, and 44.83% are transition substitution mutations. Transversion and deletion mutations were more frequent in exons than in introns of the *OCT1* gene in Bangladeshi metformin poor responder T2DM patients (Supporting Figures 1A and 1B). Among them, 13 mutations are located in the coding region and are distributed only in exons 1, 2, 6, 7, and 11; most are located in exon 7. The remaining 16 mutations are located in noncoding regions and are distributed in introns 1, 2, 4, 5, 7, 8, 9, and 10; most are in intron 1 (Supporting Figure 2).

Of the coding mutations, one novel mutation c.970G>C (p.E324Q) and six reported mutations c.181C>T (p.R61C), c.480G>C (p.L160F), c.493G>T (p.G165C), c.1022A>G (p.P341L), c.1207A>G (p.I403V), and c.1222A>G (p.M408V) are nonsynonymous SNPs (nsSNPs), whereas two reported mutations c.156T>C (p.S52S) and c.1149T>G (p.A383A) and one novel mutation c.1653C>A (p.P551P) are synonymous (Table 3). Besides, one deletion mutation (c.102_109delCATCTGTG) located in TMH1 causes frameshift (p.Ile35fs), and another two deletion mutations (c.1258_1260delATG and c.1260_1262delGAT) in TMH9 cause inframe deletion (p.M420del). On the other hand, 14 noncoding mutations are deep intronic mutations, whereas rest 2 noncoding mutations c.955-7C>T and c.1276+9_1276+16delGTAAGTTG cause splice donor variation (Table 3).

### 3.3. Deleterious Effects of Nonsynonymous and Deletion Mutations in the *OCT1* Gene

**Figure 1:**
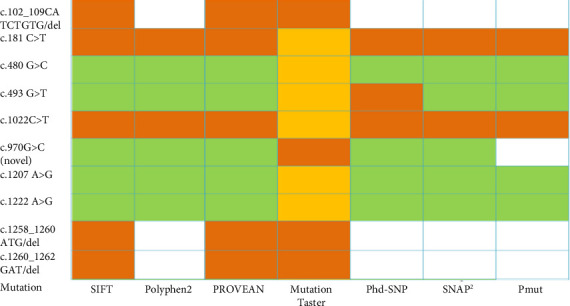
Heatmap of the prediction of deleterious impact on the OCT1 protein due to nsSNPs of the *OCT1* gene by in silico analyses. Here, the orange color indicates a damaging impact on protein, whereas the yellow color indicates slight chances of protein features being affected. Conversely, green and white colors indicate tolerable effects and no data, respectively.

The potential pathogenic effects of the nsSNPs and deletion mutations of the coding region of the *OCT1* gene on the structure and function of OCT1 protein were assessed by using 7 web-based tools (SIFT, PolyPhen-2, PROVEAN, Mutation Taster, PhD-SNP, SNAP2, and PMut). In this study, two nonsynonymous mutations c.181C>T and c.1022C>T were predicted to have potentially damaging and disease-causing effects by all 7 tools. Three deletion mutations c.102_109CATCTGTG/del, c.1258_1260ATG/del, and c.1260_1262GAT/del were predicted to have potentially damaging and disease-causing effects by three tools (Figure 1), whereas novel nsSNP was predicted to be nonpathogenic by all tools except the MutationTaster.

### 3.4. Impact of nsSNPs on Protein Stability

Protein structure stability that affects the function, activity, and regulation of biological molecules is the basic characteristic of a protein, and the free energy of protein unfolding is a key index of protein stability [40]. DDG or ΔΔ*G* (unfolding Gibbs free energy change) of the wild and mutant structures was calculated by subtracting the free energy change of the mutant protein from the free energy change of the wild protein (kcal/mol). DDG > 0 predicts high stability of the mutant protein and DDG < 0 predicts low stability [41]. The protein stability of all these nsSNPs was checked by 7 web-based tools (MUpro, INPS-MD, I-Mutant2.0, DynaMut, mCSM, SDM, and DUET). In this study, highly damaging nsSNP (predicted by previous in silico analyses) c.181C>T and another two nsSNPs c.480G>C and c.1207A>G showed negative DDG value by all seven tested tools and other nsSNPs also showed negative DDG value by five or four tools except c.1022C>T (Table 4). This mutation c.1022C>T showed a negative DDG value by 3 tools and a positive DDG value by 4 tools. Therefore, all the identified nsSNPs might decrease protein stability, and only the mutation c.1022C>T might increase the stability of the protein.

### 3.5. Alteration of mRNA Splicing due to SNPs in Coding and Noncoding Regions of the *OCT1* Gene

By analyzing with the tool Human Splice Finder (HSF), it was found that novel c.970G>C and c.1653C>A potentially altered mRNA splicing by altering an exonic enhancer site (ESE) or by activating an exonic cryptic splice donor site. High-risk mutations c.181C>T as well as c.1022C>T, two other nsSNPs c.493G>T and c.1207A>G, and deletion mutations were also altered mRNA splicing by activating or creating an exonic cryptic donor site or creating or altering ESE (Table 5). In this study, only c.480G>C and c.1222A>G did not show any impact on *OCT1* mRNA splicing. Identified intronic mutations were also studied, and it was found that only deep intronic mutation c.516-257C>T potentially altered mRNA spicing by activating the intronic cryptic donor site and creating an intronic SE site (Supporting Table 1).

### 3.6. Alterations of Interatomic Interaction in the OCT1 Protein due to nsSNPs

**Figure 2:**
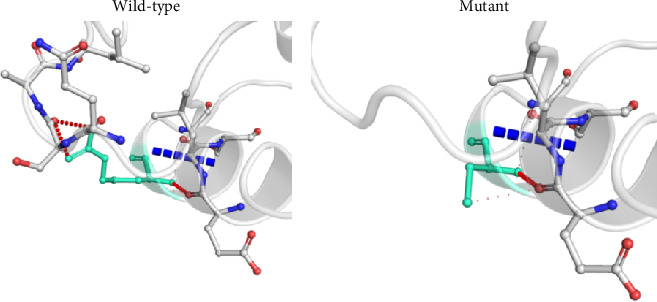
Interatomic interaction variations due to c.181C>T mutation in the *OCT1* gene. Wild-type and mutant residues are colored light green and are also represented as sticks alongside the surrounding residues which are involved in any type of interaction.

To determine the structural consequences of the altered amino acids, the protein's wild-type and mutant three-dimensional structures were retrieved from the DynaMut webserver. Not only deletion mutations but also nsSNPs have created a distinct alteration in the protein's interactions with adjacent amino acids. For instance, the loss of a hydrogen bond with the previous amino acid due to c.181C>T (p.R61C) (Figure 2) and loss of an ionic interaction due to c.1022C>T (p.P341L) mutation (Supporting Figure 3) lead to major changes in the protein's structure. These modifications could have repercussions for the protein's stability, function, and overall biological activity.

### 3.7. Genotype Analysis

The patients' genotypes of all identified novel and reported mutations were compared with general population data from the 1000 Genome database. In this study, novel mutations in the coding and noncoding regions of the *OCT1* gene were found only in Bangladeshi patients at low frequency and mostly in heterozygous form (Supporting Table 2). It was also found that the homozygous form of high-risk nsSNPs c.181C>T and c.1022C>T was more frequent in T2DM patients than in healthy controls in the Bangladeshi population. Moreover, the homozygous form of frameshift deletion mutation c.102_109CATCTGTG/del (p.Ile35GlyfsTer10) and splice region variant c.1276+9_1276+16GTAAGTTG/del were found only in patients. Also, the homozygous form of inframe deletions mutations c.1260_1262GAT/del (p.M420del) and c.1258_1260ATG/del (p.M420del) was found relatively more in patients than in controls (Supporting Table 2). In addition, the chi-square goodness-of-fit test indicated no significant deviation from HWE in the case of most of the detected SNPs in the *OCT1* gene (Supporting Table 3). This result suggested that the study population group represented the larger corresponding population.

## 4. Discussion

In drug disposition and response, genetic polymorphisms in drug transporters are being recognized as potential sources. The most commonly prescribed drug, “metformin” for T2DM patients, is transferred from the blood into the liver by the hepatic transporter OCT1. Previous studies have shown that genetic variation in *OCT1* may contribute to variation in response to metformin [12]. There are several reports regarding the genetic variation of *OCT1* and metformin response worldwide, but to the best of our knowledge, there are no reports on the genetic variation of *OCT1* with metformin response in Bangladeshi T2DM patients [42, 43]. This is the first study on the complete mutation spectrum of the *OCT1* gene in metformin poor responders among Bangladeshi T2DM patients. In this study, we identified 13 mutations in coding regions and 16 mutations in noncoding regions of the *OCT1* gene. Among the coding mutations, seven SNPs (c.181C>T, c.480G>C, c.493G>T, c.1022A>G, c.970G>C, c.1207A>G, and c.1222A>G) change amino acid sequences. Out of three deletion mutations, c.102_109delCATCTGTG caused frameshift after amino acid at 34 position and made a truncated OCT1 protein, and another two deletion mutations c.1258_1260delATG and c.1260_1262delGAT caused inframe deletion at position 420 of OCT1 protein. In our study, only two noncoding mutations (c.955-7C>T and c.1276+9_1276+16GTAAGTTG/del) are located near the splice donor site (Table 3), and the rest are deep intronic mutations. Of the identified mutations, two coding c.970G>C (p.E324Q) and c.1653C>A (p.P551P) as well as three noncoding mutations (c.412-86G>T, c.1386-3088_1386-3083GAATCA/del and c.1498+66G>T) were identified first time in Bangladeshi patients, which were not found in any other countries [23, 24].

TMHs 1, 2, 4, 5, 7, 8, 10, and 11 of human OCT1 form a large funnel-shaped substrate-binding region, which opens into the cytoplasm, and nonsynonymous SNPs in these TMHs tend to impair the transmembrane conductance of substrates by OCT1 [43, 44]. The extracellular loop between TMH1 and TMH2 has a putative role in drug binding and uptake, and the intracellular loop between TMH6 and TMH7 contains putative sites for precise regulation of OCTs functions as drug transporters [44]. In this study, identified nsSNPs, frameshift, and inframe deletion mutations are located at TMHs (1, 2, 8, and 9), large extracellular loop, large cytoplasmic TP, and C terminal cytoplasmic loop (Table 3). Therefore, identified nsSNPs might be responsible for altered metformin response and gastrointestinal side effects in this study population. For example, nsSNP c.181C>T (p.R61C) is located at a large extracellular loop that causes the loss of a hydrogen bond with the previous amino acid (Figure 2) and decreases protein stability (Table 4). Consequently, it affects transporter activity which in turn reduces the uptake of metformin and decreases the steady-state concentration of metformin [45]. Basically, this mutation is associated with metformin intolerance and causes gastrointestinal side effects [46]. This mutation was predicted to be deleterious by all seven web-based tools used in this study (Figure 1). The homozygous form of this mutation was only found in Bangladeshi T2DM patients but not observed in the healthy population from the 1000 Genome project. Another nsSNP c.480G>C (p.L160F) is located at TMH2 (Table 3) and is most common in both T2DM patients and the general population (Supporting Table 2). This mutation does not show any pathogenic effect according to our *in silico* analyses (Figure 1) and has no impact on mRNA splicing (Table 5). Another nsSNP c.1022C>T (p.P341L) located at the large extracellular loop of OCT1 has potential pathogenic effects as per several web-based tools (Figure 1). Due to this mutation, the loss of an ionic interaction leads to major changes in the protein's structure (Supporting Figure 3). It may change the mRNA splice site, too (Table 5). The homozygous form of this mutation was more frequent in patients than in the general population. This mutation is found to affect transporter activity and impair the drug uptake process [43].

Novel mutation c.970G>C (p.E324Q) is located at the large extracellular loop of OCT1. For this nsSNP, a negatively charged glutamic acid is replaced with polar uncharged amino acid glutamine acid. This nsSNP might alter the patient response to metformin not only by changing the exonic splicing enhancer site of OCT1 mRNA (Table 5) but also by decreasing the OCT1 protein stability (Table 4). Despite in silico analyses showing no mRNA splicing site alteration effect (Table 5) and no pathologic effect of nsSNP c.1222A>G (p.M408V) (Figure 1), this variant lowers the expression of OCT1 mRNA in the human liver samples [5]. This mutation was the most genotyped worldwide, and its frequency has been found to range from 15% to 80% [47]. In our study, its homozygous mutation frequency is higher in patients compared to the general population of Bangladesh (Supporting Table 2).

The deleted mutation (c.102_109CATCTGTG/del) is located at N-terminal TMH1. For this mutation, the protein is truncated after 35 amino acids. It was a very rare mutation found only in Bangladeshi T2DM patients but not in the general population. This mutation might alter mRNA splicing as well (Table 5). Another two deletion mutations cause amino acid deletion at the 420 position of the protein, and this deleted amino acid is located at TMH9. These two deletion mutations alter the splice site of the *OCT1* gene (Table 5) and might affect metformin response. In addition, previously reported 8 bases deletions in intron (c.1276+9_1276+16GTAAGTTG/del) were found only in our T2DM patients more frequently (0.321) but not in the general population. Another intronic most genotyped mutation c.1386-2964C>A was also found in the Bangladeshi population. However, this mutation has no adverse effect on this population. Moreover, InterVar tool was utilized to interpret the clinical significance of the identified genetic variants in the *OCT1* gene according to the ACMG/AMP 2015 guidelines, and the ACMG classification results mostly align with our findings, further supporting our claim (Supporting Table 4).

However, there is one limitation to this study. We collected data regarding HbA1c levels after treatment with metformin but not the level of HbA1c before treatment with metformin. As HbA1c level is a commonly used marker for glycemic control in patients with diabetes, the difference in HbA1c levels in patients before and after treatment could be used to get a clearer picture of metformin response. The aforementioned limitations signify that more research should be conducted on this topic in the future. Besides, future research could involve larger and more diverse patient cohorts from different geographical regions of Bangladesh to validate the findings and explore potential ethnic variations in *OCT1* gene mutations and their effects on metformin response.

In conclusion, we explored the clinical features as well as mutation spectrum in the coding regions and adjacent intronic consensus splice sites of the *OCT1* gene in T2DM patients who were metformin poor responders. Our study identified 5 novel mutations and 24 reported mutations in the *OCT1* gene of the patients. Among them, 3 were neutral synonymous mutations and 7 were nonsynonymous mutations, out of which 5 were damaging as per in silico analyses. In addition, our study demonstrated that frameshift and inframe deletion mutations produced truncated proteins and were potentially deleterious. Furthermore, 14 deep intronic mutations were identified, with two causing splice donor variation. Bioinformatics analysis revealed altered protein stability, mRNA splicing, and interatomic bonding due to certain mutations. That means individuals with certain mutations in the *OCT1* gene may have reduced transport of metformin into liver cells which leads to decreased effectiveness of this medication and increased gastrointestinal side effects. These findings suggest a potential role of *OCT1* gene mutations in altered metformin response in the T2DM patients of Bangladesh. These outcomes offer insights for personalized medicine and treatment optimization. In fact, this study contributes to our understanding of metformin efficacy variability and serves as a foundation for future research in this area.

## Figures and Tables

**Table 1:** Primer sequences of the *OCT1* gene (gene ID: 6580).

Primer name	Primer sequence	Target location	Product size (base pair)
OCT1-1F	ACTTGGTTGCCTTCCAGATG	Exon 1	591
OCT1-1R	TCCCAGGAACTCCCATGTTAC		

OCT1-2F	TAAGGAGGAGGAGGAAAAGAGG	Exon 2	581
OCT1-2R	ATTCCAACTGGTCATGTTCTCC		

OCT1-3F	CTGACCAGTGGCGATAATAATG	Exon 3	651
OCT1-3R	AAAGAGAGGAGGCCATTCTAGC		

OCT1-4F	GAAGGAGAAATGGGAGACACAC	Exon 4	469
OCT1-4R	CTTTGGAAGACGGCCTGTAG		

OCT1-5F	CACTGAGCAACAGCATCACC	Exon 5-6	606
OCT1-5R	TCCACCTGAGTATTCCACTGTC		

OCT1-6F	TAGGGCATTCTAAACCCAGTG	Exon 7	535
OCT1-6R	CCTAGAAATGGCACAGATGG		

OCT1-7F	TCAAGCAAGCCTCCTACCTC	Intron 8	598
OCT1-7R	TGCCTTGGCTTCTCAAAGTAC		

OCT1-8F	TTACAGCCCAGGAAACCAAG	Exon 8	556
OCT1-8R	GACCCTCTCTTGATGCTTAACC		

OCT1-9F	AGGTAGGCTCTCTGCCATTG	Exon 9	519
OCT1-9R	GCATCATCCTTGCCTTCTGT		

OCT1-10F	CTCTGACATTTCCCCAGTTATC	Exon 10	542
OCT1-10R	TCTGCCATGCACTTGAGAAC		

OCT1-11F	CTTCTTTGCTGTTTGCCATC	Exon 11	460
OCT1-11R	ACCCGATACCAATAGCACCA		

**Table 2:** Demographic, anthropometric, and biochemical parameters of the Bangladeshi study population.

Parameters	T2DM patients (*n* = 56)	Normal range
Duration of taking metformin	3.62 ± 2.97	—
Age (years)	48.07 ± 10.69	—
Sex (%)		
Male	28%	—
Female	72%	—
BMI (kg/cm^2^)	26.10 ± 3.55	18.5–24.9
FPG (mmol/L)	7.28 ± 1.98	< 6.1
PPG (mmol/L)	10.02 ± 2.76	< 7.8
HbA1c (%)	8.13 ± 2.27	< 6.5
CHO (mg/dL)	184.25 ± 35.05	< 200
TG (mg/dL)	168.18 ± 57.17	< 150
Cholesterol HDL (mg/dL)	44.43 ± 9.06	> 40
Cholesterol LDL (mg/dL)	115.48 ± 31.18	60–100
Alcohol consumption	No	—

*Note:* Here, most of the data are expressed as mean ± SD.

**Table 3:** Mutations in the *OCT1* gene of the Bangladeshi study population along with their location and functional consequences.

Mutation	Exon/intron	Coding location	Protein domain	rs number	Mutation type	Functional consequence	Amino acid change
CATCTGTG/del	Exon 1	c.102_109	TMH1	rs776450090	Deletion	Frameshift variant	Ile35fs
C>T	Exon 1	c.181	Large extracellular TP	rs122083571	Ts	Nonsynonymous variant	R61C
T>C	Exon 1	c.156	Large extracellular TP	rs1867351	Ts	Synonymous variant	S52S
T>C	Intron 1	c.412-207	—	rs9457841	Ts	Intron variant	—
A>G	Intron 1	c.412-174	—	rs370897802	Ts	Intron variant	—
G>T	Intron 1	c.412-86	—	Novel	Tr	Intron variant	—
T>G	Intron 1	c.412-43	—	rs4646272	Tr	Intron variant	—
G>C	Exon 2	c.480	TMH2	rs683369	Tr	Nonsynonymous variant	L160F
G>T	Exon 2	c.493	TMH2	rs201942835	Tr	Nonsynonymous variant	G165C
C>T	Intron 2	c.516-26	—	rs45584532	Ts	Intron variant	—
C>T	Intron 2	c.516-257	—	rs4646275	Ts	Intron variant	—
G>C	Intron 4	c.840-37	—	rs773045134	Tr	Intron variant	—
G>A	Intron 5	c.955-61	—	rs2282142	Ts	Intron variant	—
C>T	Intron 5	c.955-7	—	rs7762846	Ts	Splice region variant	—
C>T	Exon 6	c.1022	Large cytoplasmic TP	rs2282143	Ts	Nonsynonymous variant	P341L
G>C	Exon 6	c.970	Large cytoplasmic TP	Novel	Tr	Nonsynonymous variant	E324Q
T>G	Exon 7	c.1149	TMH8	rs762648108	Tr	Synonymous variant	A383A
A>G	Exon 7	c.1207	TMH9	rs188898744	Ts	Nonsynonymous variant	I403V
A>G	Exon 7	c.1222	TMH9	rs628031	Ts	Nonsynonymous variant	M408V
ATG/del	Exon 7	c.1258_1260	TMH9	rs202220802	Deletion	Inframe deletion	M420del
GAT/del	Exon 7	c.1260_1262	TMH9	rs72552763	Deletion	Inframe deletion	M420del
GTAAGTTG/del	Intron 7	c.1276+9-1276+16	—	rs35854239	Deletion	Splice donor variant	—
T>A	Intron 8	c.1386-3273	—	rs9347388	Tr	Intron variant	—
C>A	Intron 8	c.1386-2964	—	rs622342	Tr	Intron variant	—
GAATCA/del	Intron 8	c.1386-3088_1386-3083	—	Novel	Deletion	Intron variant	—
G>T	Intron 9	c.1498+66	—	Novel	Tr	Intron variant	—
C>T	Intron 9	c.1498+43	—	rs2297374	Ts	Intron variant	—
T>C	Intron 10	c.1599-21	—	rs622591	Ts	Intron variant	—
C>A	Exon 11	c.1653	Cytoplasmic TP	Novel	Tr	Synonymous variant	P551P

Abbreviations: TMH = transmembrane helical, TP = topological domain, Tr = transvertion substitution, Ts = transition substitution.

**Table 4:** Protein stability test by different web-based tools.

Mutations	DDG						
	MUpro	INPS-MD	I-mutant2.0	DynaMut	mCSM	SDM	DUET
c.181C>T (p.R61C)	−0.61123093	−0.0619008	−0.28	−0.312	−0.04	−0.07	−0.06
c.480G>C (p.L160F)	−1.1017082	−0.311019	−0.19	−0.248	−1.218	−0.69	−1.267
c.493G>T (p.G165C)	−0.43919442	1.26756	−1.39	0.079	−0.799	0.45	−0.397
c.1022C>T (p.P341L)	−0.13970588	−1.02966	0.08	0.947	−0.328	1.57	0.421
c.970G>C (p.E324Q)	−1.1889861	−0.192615	−0.05	0.418	0.279	−0.69	0.453
c.1207A>G (p.I403V)	−0.48786515	−0.145814	−0.65	−0.611	−1.244	−2.79	−1.615
c.1222A>G (p.M408V)	−0.87796438	0.196276	−0.26	0.010	−0.767	−0.07	−0.265

**Table 5:** Potential impact of *OCT1* coding region mutations on mRNA splicing by HSF.

SNP	Predicted signal	Interpretation
c.181C>T (p.R61C)	Creation of an ESS site and alteration of an ESE site	Potential alteration of splicing
c.480G>C (p.L160F)	NS	No impact on splicing
c.493G>T (p.G165C)	Creation of an ESS site	Potential alteration of splicing
c.1022C>T (p.P341L)	Alteration of an ESE site	Potential alteration of splicing
c.970G>C (p.E324Q)	Alteration of an ESE site	Potential alteration of splicing
c.1207A>G (p. I403V)	Alteration of an ESE site	Potential alteration of splicing
c.1222A>G (p.M408V)	NS	No impact on splicing
c.1653C>A (p.P551P)	Activation of an cryptic donor site and alteration of an ESE site	Potential alteration of splicing
c.102_109CATCTGTG/del (p.Ile35GlyfsTer10)	Activation of an cryptic donor site, creation of an ESS site, and alteration of an ESE site	Potential alteration of splicing
c.1258_1260ATG/del (p.M420del)	Creation of an ESS and alteration of an ESE site	Potential alteration of splicing
c.1260_1262GAT/del (p.M420del)	Creation of an ESS site and alteration of an ESE site	Potential alteration of splicing

Abbreviations: ESE = exonic splicing enhancers, ESS = exonic splicing silencers, NS = nonsignificant.

## Data Availability

The data that support the findings of this study are available in the supporting information of this article.

## Supporting Information

Supporting Table 1: Prediction of alternative mRNA splicing effect of *OCT1* noncoding region mutation by HSF. Supporting Table 2: Genotype frequency of *OCT1* gene mutations in the study population and general population of Bangladesh. Supporting Table 3: Chi-square goodness-of-fit test for Hardy–Weinberg equilibrium status analysis of the study population group. Supporting Table 4: Clinical interpretation of identified genetic variants of the *OCT1* gene in the study population by ACMG/AMP 2015 guideline.

Supporting Figure 1: Percent distribution of mutation types found in the *OCT1* gene of metformin poor responders among Bangladeshi T2DM patients. Supporting Figure 2: Frequency distribution of mutations at different regions of the *OCT1* gene in Bangladeshi T2DM patients with poor response to metformin. Supporting Figure 3: Loss of ionic interaction due to c.1022C>T mutation in the *OCT1* gene. Wild-type and mutant residues are colored light green and are also represented as sticks alongside the surrounding residues which are involved in any type of interaction.
