# Supplementary material for: Insights Into Genetic Variations of the OCT1 Gene in Metformin Poor Responders Among Bangladeshi Type 2 Diabetic Patients
Source: Adv Pharmacol Pharm Sci. 2025 Jan 29;2025:8568658. doi: 10.1155/adpp/8568658 (PMC11824854; doi:10.1155/adpp/8568658)
Supplement: Supporting Information — Additional supporting information can be found online in the Supporting Information section. [file 8568658.f1.docx]

SUPPORTING TABLE 1: Prediction of alternative mRNA splicing effect of *OCT1* non-coding region mutation by HSF.

| Mutations | Reported ID  of mutations | Predicted signal | Interpretation |
| --- | --- | --- | --- |
| c.412-207T>C | rs9457841 | Creation of an intronic SE site. | No impact on splicing |
| c.412-174A>G | rs370897802 | Creation of an intronic SE site. | No impact on splicing |
| c.412-86G>T | Novel | No significant impact on splicing signals | No impact on splicing |
| c.412-43T>G | rs4646272 | No significant impact on splicing signals | No impact on splicing |
| c.516-26C>T | rs45584532 | Alteration of an intronic SS site | No impact on splicing |
| c.516-257C>T | rs4646275 | Activation of an intronic cryptic donor site and creation of an intronic SE site | Potential alteration of splicing |
| c.840-37G>C | rs773045134 | Creation of an intronic SE site | No impact on splicing |
| c.955-61G>A | rs2282142 | Creation of an intronic SE site | No impact on splicing |
| c.955-7C>T | rs7762846 | No significant impact on splicing signals | No impact on splicing |
| c.1276+9_1276+16 GTAAGTTG/ del | rs866909435 | Alteration of an intronic SS site. | No impact on splicing |
| c.1386-3273T>A | rs9347388 | Alteration of an intronic SS site. | No impact on splicing |
| c.1386-2964C>A | rs622342 | No significant impact on splicing signals | No impact on splicing |
| c.1386-3088_1386-3083GAATCA/del | Novel | Alteration of an intronic SS site and creation of an intronic SE site | No impact on splicing |
| c.1498+66G>T | Novel | Alteration of an intronic SS site. | No impact on splicing |
| c.1498+43C>T | rs2297374 | No significant impact on splicing signals | No impact on splicing |
| c.1599-21C>T | rs622591 | No significant impact on splicing signals | No impact on splicing |

*Here,* SE= splicing enhancer, SS= splicing silencer.

SUPPORTING TABLE 2: Genotype frequency of *OCT1* gene mutations in the study population and general people of Bangladesh.

| Mutation location within the gene | Mutation | rs Number | Mutant Genotype frequency | | | |
| --- | --- | --- | --- | --- | --- | --- |
|  |  |  | Bangladeshi T2DM patients in this study (n=56) | | Bangladeshi population in 1000 Genomes Project (n=86) | |
|  |  |  | Hom | Het | Hom | Het |
| Coding region of *OCT1* gene | c.102_109CATCTGTG/del  (p.Ile35GlyfsTer10) | rs776450090 | 0.018  (1) | - | NR | NR |
|  | c.181C>T  (p.R61C) | rs122083571 | 0.018  (1) | 0.036  (2) | - | 0.035 (3) |
|  | c.156T>C  (p.S52S) | rs1867351 | 0.036  (2) | 0.286  (16) | 0.023 (2) | 0.326 (28) |
|  | c.480G>C  (p.L160F) | rs683369 | 0.607  (34) | 0.286  (16) | 0.733 (63) | 0.244 (21) |
|  | c.493G>T  (p.G165C) | rs201942835 | - | 0.071  (4) | - | 0.047 (4) |
|  | c.1022C>T  (p.P341L) | rs2282143 | 0.018  (1) | 0.179  (10) | 0.012 (1) | 0.256 (22) |
|  | c.970G>C  (p.E324Q) | novel | - | 0.036  (2) | NR | NR |
|  | c.1149T>G  (A383A) | rs762648108 | - | 0.018  (1) | NR | NR |
|  | c.1207A>G  (p.I403V) | rs188898744 | - | 0.071  (4) | - | 0.070 (6) |
|  | c.1222A>G  (p.M408V) | rs628031 | 0.429  (24) | 0.232  (13) | 0.360 (31) | 0.500 (43) |
|  | c.1258_1260ATG/del (p.M420del) | rs72552763 | 0.018  (1) | - | 0.012 (1) | 0.221 (19) |
|  | c.1260_1262GAT/del  (p.M420del) | rs72552763 | 0.089  (5) | - | 0.012 (1) | 0.221 (19) |
|  | c.1653C>A  (p.P551P) | novel | - | 0.018  (1) | NR | NR |
| Non-Coding region of *OCT1* gene | c.412-207T>C | rs9457841 | 0.107  (6) | 0.393  (22) | 0.163 (14) | 0.512 (44) |
|  | c.412-174A>G | rs370897802 | - | 0.036  (2) | - | 0.012 (1) |
|  | c.412-86G>T | novel | - | 0.071  (4) | NR | NR |
|  | c.412-43T>G | rs4646272 | 0.036  (2) | 0.143  (8) | 0.070 (6) | 0.407 (35) |
|  | c.516-26C>T | rs45584532 | - | 0.107  (6) | 0.012 (1) | 0.105 (9) |
|  | c.516-257C>T | rs4646275 | - | 0.036  (2) | 0.012 (1) | 0.314 (27) |
|  | c.840-37G>C | rs773045134 | - | 0.018  (1) | - | NR |
|  | c.955-61G>A | rs2282142 | 0.018  (1) | 0.196  (11) | 0.012 (1) | 0.256 (22) |
|  | c.955-7C>T | rs7762846 |  | 0.160  (9) | 0.012 (1) | 0.105 (9) |
|  | c.1276+9_1276+16 GTAAGTTG/del | rs35854239 | 0.321  (18) | - | NR | NR |
|  | c.1386-3273T>A | rs9347388 | 0.143  (8) | 0.464  (26) | 0.128 (11) | 0.419 (36) |
|  | c.1386-2964C>A | rs622342 | 0.429  (24) | 0.393  (22) | 0.593 (51) | 0.372 (32) |
|  | c.1386-3088_1386  -3083GAATCA/del | novel | 0.018  (1) | - | NR | NR |
|  | c.1498+66G>T | novel | - | 0.036  (2) | NR | NR |
|  | c.1599-21C>T | rs622591 | 0.429  (12) | 0.179  (10) | 0.140 (12) | 0.581 (50) |
|  | c.1498+43C>T | rs2297374 | 0.179  (10) | 0.500  (28) | 0.128 (11) | 0.430 (37) |

*Here,* Hom = Homozygous, Het = Heterozygous, NR = No records.

SUPPORTING TABLE 3: Chi-square goodness of fit test for Hardy-Weinberg equilibrium status analysis of the study population group.

| Mutation | Frequency of A (p) | Frequency of 𝑎 (q) | Expected genotype frequencies | χ 2 | Chi-square test result | Hardy-Weinberg equilibrium status |
| --- | --- | --- | --- | --- | --- | --- |
| c.181C>T | 0.964 | 0.036 | AA: 0.929 Aa: 0.069 aa: 0.0013 | 13.27 | Chi-square value (13.27) is greater than the critical value (3.841). The differences between the observed and expected genotype frequencies are statistically significant | The studied group is not in Hardy-Weinberg equilibrium. |
| c.156T>C | 0.8214 | 0.1786 | AA: 0.6747 Aa: 0.2933 aa: 0.0319 | 0.037 | Chi-square value (0.037) is much less than the critical value (3.841). The observed genotype frequencies are not significantly different from the expected frequencies. | The studied group is in Hardy-Weinberg equilibrium. |
| c.480G>C | 0.25 | 0.75 | AA: 0.0625 Aa: 0.375 aa: 0.5625 | 3.18 | Chi-square value (3.18) is less than the critical value (3.841). The observed genotype frequencies are not significantly different from the expected frequencies | The studied group is in Hardy-Weinberg equilibrium. |
| c.493G>T | 0.964 | 0.036 | AA: 52.07 Aa: 3.86 aa: 0.07 | 0.077 | Chi-square value (0.77) is less than critical value (3.841). There is no significant difference between the observed and expected genotype frequencies. | The studied group is likely in Hardy-Weinberg equilibrium. |
| c.1022C>T | 0.893 | 0.107 | AA: 44.8 Aa: 10.7 aa: 0.6 | 0.313 | Chi-square value (0.313) is much less than critical value (3.841). The observed genotype frequencies are not significantly different from the expected frequencies. | The studied group is in Hardy-Weinberg equilibrium. |
| c.970G>C | 0.982 | 0.018 | AA: 0.964 Aa: 0.035 aa: 0.0003 | 0.018 | Chi-square value (0.018) is much less than critical value (3.841). The observed genotype frequencies are not significantly different from the expected frequencies. | The studied group is in Hardy-Weinberg equilibrium. |
| c.1149T>G | 0.991 | 0.009 | AA: 0.982 Aa: 0.0178 aa: 0.00008 | 0 | Chi-square value (0) much less than the critical value (3.841). The observed genotype frequencies are not significantly different from the expected frequencies. | The studied group is in Hardy-Weinberg equilibrium. |
| c.1207A>G | 0.964 | 0.036 | AA: 0.929 Aa: 0.069 aa: 0.0013 | 0.074 | Chi-square value (0.074) is less than the critical value (3.841). The observed genotype frequencies are not significantly different from the expected frequencies. | The studied group is in Hardy-Weinberg equilibrium. |
| c.1222A>G | 0.455 | 0.545 | AA: 0.207 Aa: 0.496 aa: 0.297 | 15.87 | Chi-square value (15.87) is much larger than critical value (3.841). There is significant difference between the observed and expected genotype frequencies. | The studied group is not in Hardy-Weinberg equilibrium. |
| c.1653C>A | 0.991 | 0.009 | AA: 0.982 Aa: 0.0178 aa: 0.00008 | 0 | Chi-square value (0) much less than critical value (3.841). The observed genotype frequencies are not significantly different from the expected frequencies. | The studied group is in Hardy-Weinberg equilibrium. |
| c.412-207  T>C | 0.732 | 0.268 | AA: 0.536 Aa: 0.392 aa: 0.072 | 1 | Chi-square value (1) is less than critical value (3.841). The differences between observed and expected frequencies are not statistically significant. | The studied group is in Hardy-Weinberg equilibrium. |
| c.412-174  A>G | 0.982 | 0.018 | AA: 0.964 Aa: 0.035 aa: 0.0003 | 0.018 | Chi-square value (0.018) is much less than critical value (3.841). The observed genotype frequencies are not significantly different from the expected frequencies. | The studied group is in Hardy-Weinberg equilibrium. |
| c.412-86  G>T | 0.964 | 0.036 | AA: 0.929 Aa: 0.069 aa: 0.0013 | 0.074 | Chi-square value (0.074) is less than critical value (3.841). The observed genotype frequencies are not significantly different from the expected frequencies. | The studied group is in Hardy-Weinberg equilibrium. |
| c.412-43  T>G | 0.893 | 0.107 | AA: 0.798 Aa: 0.191 aa: 0.011 | 3.986 | Chi-square value (3.986) is slightly greater than critical value (3.841). The difference between observed and expected frequencies is statistically significant | The studied group might not be in Hardy-Weinberg equilibrium. |
| c.516-26  C>T | 0.946 | 0.054 | AA: 0.895 Aa: 0.102 aa: 0.003 | 0.216 | Chi-square value (0.216) is much smaller than critical value (3.841). The difference between observed and expected frequencies is not statistically significant | The studied group is in Hardy-Weinberg equilibrium. |
| c.516-257  C>T | 0.982 | 0.018 | AA: 0.964 Aa: 0.035 aa: 0.0003 | 0.018 | Chi-square value (0.018) is much less than critical value (3.841). The observed genotype frequencies are not significantly different from the expected frequencies. | The studied group is in Hardy-Weinberg equilibrium. |
| c.840-37  G>C | 0.991 | 0.009 | AA: 0.982 Aa: 0.0178 aa: 0.00008 | 0 | Chi-square value (0) much less than critical value (3.841). The observed genotype frequencies are not significantly different from the expected frequencies. | The studied group is in Hardy-Weinberg equilibrium. |
| c.955-61  G>A | 0.884 | 0.116 | AA: 0.781 Aa: 0.205 aa: 0.013 | 0.153 | Chi-square value (0.153) is much smaller than critical value (3.841). The differences between observed and expected frequencies are not statistically significant | The studied group is in Hardy-Weinberg equilibrium. |
| c.955-7C>T | 0.92 | 0.08 | AA: 0.846 Aa: 0.147 aa: 0.0064 | 0.481 | Chi-square value (0.481) is much smaller than critical value (3.841). The differences between observed and expected genotype frequencies are not statistically significant | The studied group is in Hardy-Weinberg equilibrium. |
| c.1386-3273  T>A | 0.625 | 0.375 | AA: 0.391 Aa: 0.469 aa: 0.141 | 0.005 | Chi-square value (0.005) is much smaller than critical value (3.841). The differences between observed and expected frequencies are not statistically significant | The studied group is in Hardy-Weinberg equilibrium. |
| c.1386-2964  C>A | 0.375 | 0.625 | AA: 0.1406 Aa: 0.4688 aa: 0.3906 | 1.47 | Chi-square value (1.47) is much less than critical value (3.841). The observed genotype frequencies are not significantly different from the expected frequencies. | The studied group is in Hardy-Weinberg equilibrium. |
| c.1498+66  G>T | 0.982 | 0.018 | AA: 0.964 Aa: 0.035 aa: 0.0003 | 0.018 | Chi-square value (0.018) is much less than critical value (3.841). The observed genotype frequencies are not significantly different from the expected frequencies. | The studied group is in Hardy-Weinberg equilibrium. |
| c.1599-21  C>T | 0.696 | 0.304 | AA: 0.484 Aa: 0.424 aa: 0.092 | 19.1 | Chi-square value (19.10) is greater than critical value (3.841). The differences between observed and expected frequencies are statistically significant. | The studied group is not in Hardy-Weinberg equilibrium. |
| c.1498+43  C>T | 0.571 | 0.429 | AA: 0.326 Aa: 0.490 aa: 0.184 | 0.024 | Chi-square value (0.024) is much less than critical value (3.841). The difference between observed and expected frequencies is not statistically significant. | The studied group is in Hardy-Weinberg equilibrium. |

*Here,* *the degrees of freedom (df) = 1. Therefore, the critical value at 𝛼 =0.05 is 3.841.*

SUPPORTING TABLE 4: Clinical interpretation of identified genetic variants of *OCT1* gene in the study population by ACMG/AMP 2015 guideline.

| Genetic Varients | Applied ACMG Criteria | ACMG Classification |
| --- | --- | --- |
| c.102_109CATCTGTG/del | PS4 PM2 PM4 PM6 PP3 | Likely Pathogenic |
| c.181C>T | PS4 PM2 PM6 PP3 | Likely Pathogenic |
| c.156T>C | PM6 BS2 BP4 BP7 | Likely Benign |
| c.480G>C | BS1 BS2 BP4 BP7 | Likely Benign |
| c.493G>T | PM2, PP3, BP4 | Uncertain Significance |
| c.1022C>T | PM1 PM2 PM6 PP3 PP6 | Likely Pathogenic |
| c.970G>C | PM1 PM2 PM5 PM6 PP3 BP4 | Likely Pathogenic |
| c.1149T>G | BP4 BP7 | Likely Benign |
| c.1207A>G | PM1 PM2 PM6 PP3 BP4 | Likely Pathogenic |
| c.1222A>G | PS3 BS1 BS3 BP4 BP7 | Likely Benign |
| c.1258_1260ATG/del | PM1 PM4 PP3 | Uncertain Significance |
| c.1260_1262GAT/del | PM1 PM4 PP3 | Uncertain Significance |
| c.1653C>A | PM2 BP4 | Uncertain Significance |
| c.412-207T>C | BS2 BP4 BP7 | Likely Benign |
| c.412-174A>G | BP4 BP7 | Likely Benign |
| c.412-86G>T | PM2 BP4 BP7 | Likely Benign |
| c.412-43T>G | BS2 BP4 BP7 | Likely Benign |
| c.516-26C>T | BP4 BP7 | Likely Benign |
| c.516-257C>T | BS2 BP4 | Likely Benign |
| c.840-37G>C | BP4 | Uncertain Significance |
| c.955-61G>A | BS2 BP4 | Likely Benign |
| c.955-7C>T | BS2 BP4 | Likely Benign |
| c.1276+9_1276+16GTAAGTTG/del | PM2 PP3 | Uncertain Significance |
| c.1386-3273T>A | BS2 BP4 | Likely Benign |
| c.1386-2964C>A | BS2 BP4 | Likely Benign |
| c.1386-3088_1386-3083GAATCA/del | PM2 BP4 | Uncertain Significance |
| c.1498+66G>T | PM2 BP4 | Uncertain Significance |
| c.1599-21C>T | BS2 BP4 | Likely Benign |
| c.1498+43C>T | BS2 BP4 | Likely Benign |


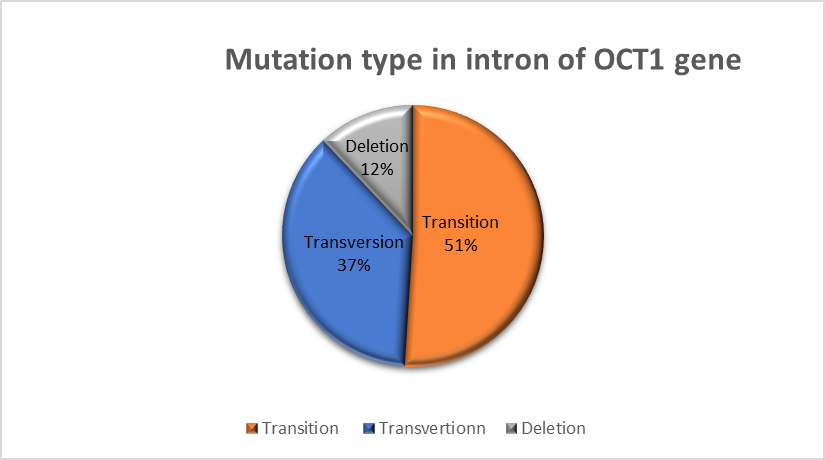


**(a)**


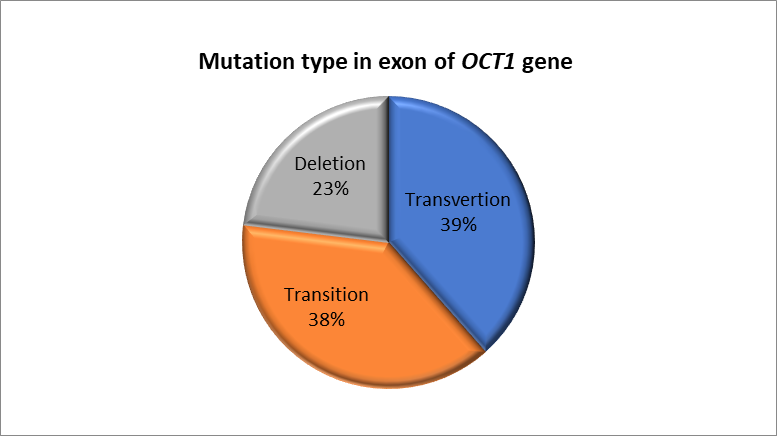


**(b)**

SUPPORTING FIGURE 1: Percent distribution of mutation types found in *OCT1* gene of metformin poor responders among Bangladeshi T2DM patients. **(a)** Mutation types in exons of *OCT1* gene. **(b)** Mutation types in introns of *OCT1* gene.

SUPPORTING FIGURE 2**:** Frequency distribution of mutations at different regions of *OCT1* gene in Bangladeshi T2DM patients with poor response to metformin.

SUPPORTING FIGURE 3: Loss of ionic interaction due to c.1022C>T mutation in *OCT1* gene. Wild-type and mutant residues are colored light green and are also represented as sticks alongside the surrounding residues which are involved in any type of interaction.
